# Enhanced West Nile Virus Circulation in the Emilia-Romagna and Lombardy Regions (Northern Italy) in 2018 Detected by Entomological Surveillance

**DOI:** 10.3389/fvets.2020.00243

**Published:** 2020-05-05

**Authors:** Mattia Calzolari, Paola Angelini, Luca Bolzoni, Paolo Bonilauri, Roberto Cagarelli, Sabrina Canziani, Danilo Cereda, Monica Pierangela Cerioli, Mario Chiari, Giorgio Galletti, Giovenale Moirano, Marco Tamba, Deborah Torri, Tiziana Trogu, Alessandro Albieri, Romeo Bellini, Davide Lelli

**Affiliations:** ^1^Istituto Zooprofilattico Sperimentale Della Lombardia e Dell'Emilia-Romagna, B. Ubertini, Brescia, Italy; ^2^Regional Health Authority of Emilia-Romagna, Bologna, Italy; ^3^Regional Health Authority of Lombardy, Milan, Italy; ^4^Cancer Epidemiology Unit-CERMS, Department of Medical Sciences, University of Turin and CPO-Piemonte, Turin, Italy; ^5^Centro Agricoltura Ambiente “G. Nicoli”, Crevalcore, Italy

**Keywords:** West Nile virus, *Culex pipiens*, surveillance, infection rate, temperature, one-health

## Abstract

With several human cases reported annually since 2008 and the unapparent risk of infection of blood donors, the West Nile virus (WNV) is emerging as an important health issue in Europe. Italy, as well as other European countries, experienced a recrudescence of the virus circulation in 2018, which led to an increased number of human cases. An integrated surveillance plan was activated in the Emilia-Romagna and Lombardy regions (Northern Italy) since 2008 in order to monitor the intensity and timing of WNV circulation. A fundamental part of this plan consists in entomological surveillance. In 2018, the surveillance plan made it possible to collect 385,293 mosquitoes in 163 stations in the two Regions. In total 269,147 *Culex* mosquitoes were grouped into 2,337 pools and tested for WNV, which was detected in 232 pools. Circulation started in the central part of the Emilia-Romagna region in the middle of June, about one month before the previous seasons. Circulation suddenly expanded to the rest of the region and reached the Lombardy region in the middle of July. WNV circulated more intensively in the eastern part of the surveyed area, as confirmed by the highest number of human cases. A relationship between the number of mosquitoes collected and the virus incidence emerged, but the data obtained highlighted that the probability of detecting the virus in a given site was less than expected with a higher number of collected mosquitoes. A significant relationship was observed between the temperature recorded one week before the sampling and the number of collected mosquitoes, as well as between the estimated number of WNV-positive mosquitoes and the temperature recorded two weeks before the sampling. The two weeks delay in the influence of temperature on the positive mosquitoes is in line with the time of the virus extrinsic incubation in the mosquito. This finding confirms that temperature is one of the principal drivers in WNV mosquito infection. The surveillance system demonstrated the ability to detect the virus circulation early, particularly in areas where circulation was more intense. This allowed evaluating the effect of mosquito abundance and weather factors on virus circulation.

## Introduction

The West Nile Virus (WNV) is a flavivirus that circulates in the environment among birds and mosquitoes, but can infect other animals such as equids and humans as dead-end hosts. In humans, virus infection is generally, asymptomatic, but about 20% of infected subjects can develop a febrile illness, which can evolve into a neuro-invasive disease (WNND) in a minority of cases (less than 1% of infected subjects). In addition to symptomatic human cases, the presence of infected, but asymptomatic, blood and organ donors is a primary health issue (1).

After sporadic reports in the '90s in Romania (2) and in Italy (3), as from 2008, the circulation of the virus was recorded at a different extent every season in Europe. In 2018, an alarming recrudescence of the virus circulation was recorded in Europe (4). This recrudescence was also noticed in Northern Italy, including the Emilia-Romagna and Lombardy regions, where an interdisciplinary surveillance plan targeting WNV was set up in 2008 and implemented over the years (5–10).

The plan focuses on the early detection and the pinpointing of the virus circulation area, including those areas where the virus did not circulate the previous season. Over time, the system has shown that it is capable of WNV circulation's early detection at provincial scale (6) and it is economically sustainable (11). Moreover, the plan was able to detect other arboviruses circulating in the surveyed area (12).

The plan has a one-health approach in order to maximize early detection, involving searching for the virus in humans, horses, birds, and mosquitoes. Entomological surveillance is a fundamental part of the plan, characterized by regular scheduling of samplings, and the precise geographic characterization of obtained samples. This permits a fine characterization of the virus circulation on the surveyed area. In this work, we characterize the extraordinary WNV circulation observed in 2018, utilizing data from entomological surveillance obtained in the Emilia-Romagna and Lombardy regions. In particular, we describe the relationship between WNV detection and the abundance of collected mosquitoes, as well as the influence of weather conditions, such as weekly maximum temperatures and number of wet days, on the virus circulation in mosquitoes.

## Materials and Methods

### Surveyed Area

The surveyed area included the plan area of the Emilia-Romagna and Lombardy regions. This is a large portion of Italy's main plain, the Pianura Padana (or Pianura Padano-Veneta), which includes about 24,000 km^2^ (51.3%) of the total area of 44,700 km^2^. This area encompasses about 10 million inhabitants, with many urban areas, including two of the biggest Italian cities (Milan and Bologna), as well as several important industrial districts. This area has a relevant livestock patrimony and is one of the more important agricultural areas in Italy; distinctive cultivations such as rice fields, vineyards and orchards are highly represented in specific areas.

In general, a strong anthropic modification, with the abundant presence of industrial and urban settlements, characterizes the surveyed area. The rural part of the territory is connoted by an intensive agriculture and animal husbandry, with few hedges, rare scattered trees, and a dense irrigation network. Natural areas are rare, mainly represented by river borders, characterized by riparian vegetation, or re-naturalized areas. The eastern part of the surveyed area ends in the Adriatic Sea, where some of the largest wetlands in Europe are present (Valli di Comacchio and the Po River Delta); pinewood and typical Mediterranean vegetation can be found in this zone.

**Figure 1:**
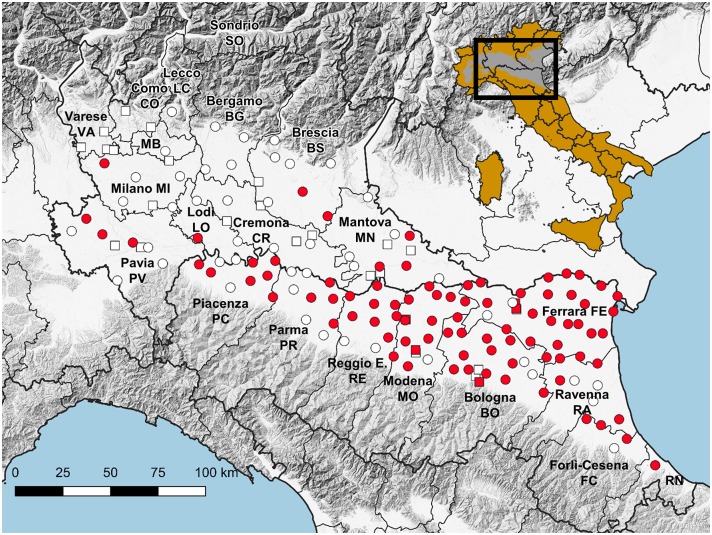
Map showing the location of traps working throughout the season (circles) and for part of the season (squares), with reference to WNV detections (red), and the reference of the surveyed area on a map of Italy in which the Pianura Padano-Veneta is depicted in gray.

Trapping sites, with different densities, were selected to cover the entire plain area of the two regions: 95 traps in Emilia-Romagna (surveillance grid of 110 km^2^) and 39 traps in Lombardy (surveillance grid of 400 km^2^) (Table 1, Figure 1). These traps worked regularly throughout the surveillance season (seasonal traps). Surveillance was focused on rural areas, semi-natural, rural, or peri-urban locations in Emilia-Romagna, and farms in Lombardy. A supplementary effort was carried out by activating 23 specific traps along the surveillance season in Lombardy, and 9 supplementary traps, which worked once (on August 10th), among selected urban areas in Emilia-Romagna.

**Table 1:** Features of surveillance in the two surveyed regions.

	**Emilia-Romagna**	**Lombardy**
Number of seasonal traps	95	39
Number of extra traps	9	23
Maximum number of mosquitoes per pool	200	100
Maximum number of mosquitoes tested per sampling	all sampled	1000
Start date	June 12	June 4
End date	October 16	October 25

### Mosquito Analysis

We collected mosquitoes in fixed geo-referenced stations using attractive traps baited by carbon dioxide, working overnight, roughly from 5:00 p.m. to 9:00 a.m. Every site regularly included in the surveillance was sampled every fortnight. Mosquitoes were identified using morphological characteristics according to the classification key of Severini et al. (13). Due to the used trap model, baited with carbon dioxide, we collected almost exclusively host seeking females, the catching of males was very rare. Identified mosquitoes were counted and pooled according to date, location, and species. The maximum number of specimens per pool was 200 in Emilia-Romagna and 100 in Lombardy. A maximum of 1,000 specimens per species was tested for every sample in Lombardy. Pooled mosquitoes belonging to the genus *Culex*, and selected pools of other species, were prepared and submitted to biomolecular tests as described elsewhere (14).

In brief, pooled mosquitoes were ground by a vortex mixer with 2-3 4.5-mm-diameter copper-plated round balls (H&N Sport, Münden, Germany) in 2 ml of PBS. After centrifugation, 200 μL aliquots were collected and submitted to biomolecular analysis, for extracting and retrotranscribing RNA. We tested samples by a WNV real-time RT-PCR, according to the method described by Tang et al. (15). This was confirmed by the protocol of Del Amo et al. (16) and by an universal PCR protocol for the identification of flaviviruses (17).

We estimated the number of positive mosquitoes for every site and the day of sampling utilizing the Maximal Likelihood Estimation (MLE) of the PooledInfRate 4.0 excel add-in (18), multiplying the MLE of the infectious rate for the number of collected mosquitoes, and rounding up the obtained result. MLE cannot be estimated when all sample pools test positive. In this case, we approximated the number of infected mosquitoes using the Minimum Infection Rate (MIR), assuming that only one positive mosquito was present in each pool.

We tested the relationship between the fraction of infected mosquitoes collected in each province, with at least two working seasonal traps, and the incidence of WNND cases in the same province through a linear model. Additionally, we checked the hypothesis of normality in the distribution of model residuals through the Shapiro-Wilk test.

### Mosquito Abundance and WNV Circulation

We tested the effect of mosquito abundance in a given area (using the catches per trap as a proxy of abundance) on the probability of WNV circulation in the same area in 2018. To guarantee comparable catching efforts, considering the start/end of trapping seasons differed among traps, we used the total number of mosquitoes caught in each trap in July and August (for a total of four sampling sessions) as a measure of mosquito abundance. Traps with less than four sampling sessions in the July-August time window were excluded from the analyses. We built a generalized linear model (GLM) with binomial error distribution and logit link function to estimate the probability of observing a WNV-positive trap in 2018, using the observed number of mosquitoes caught in each trap and the region where the trap was deployed as explanatory variables. In this model, the effect on the GLM of caught mosquitoes (namely, *M*_*OBS*_) represents the marginal increase in the probability of observing a WNV-positive trap due to an additional mosquito caught (in the logit link).

To test the effect of mosquito abundance on WNV circulation, we built a null model where the probability of each mosquito to test WNV-positive only depends on the region where the mosquito was caught and not on the abundance of mosquitoes in the trap area. These probabilities, namely *p*_*NULL*_*ER*_ and *p*_*NULL*_*LO*_, were estimated by maximizing the likelihood of obtaining the observed pattern of positive traps in 2018, given the number of mosquitoes caught in each trap in Emilia-Romagna and Lombardy, respectively. We then randomly generated 10,000 synthetic samples where we assigned a WNV-positive/negative status to each mosquito caught in Emilia-Romagna [resp. Lombardy] with probabilities *p*_*NULL*_*ER*_ [resp. *p*_*NULL*_*LO*_]. Analogously to the observed sample, we estimated for each synthetic sample the effect of the number of mosquitoes caught on the probability of observing a WNV-positive trap (namely, *M*_*NULL*_) through GLMs. Thus, we generated a distribution of the estimated *M*_*NULL*_ which represents the null hypothesis where mosquito abundance in a given area does not affect the probability of each single mosquito testing WNV-positive. We then tested the observed effect, *M*_*OBS*_, against the distribution of *M*_*NULL*_. If *M*_*OBS*_ is significantly higher [resp. smaller] than *M*_*NULL*_ (i.e., it falls outside the 95% range of the *M*_*NULL*_ distribution), then mosquito abundance provides an amplification [resp. dilution] effect on WNV circulation. Analyses were performed with R software.

In order to spatially characterize mosquito abundance, we interpolated the number of mosquitoes sampled in each seasonal trap between July-August (expressed as a natural logarithm) on a map, using the inverse distance weighting method (IDW). The virus circulation's spatial pattern was obtained using positive trap location with the kernel density estimation (KDE) method, with the bandwidth size of 15 km. The area of more intense WNV circulation was estimated through the 50% volume contour of the obtained WNV KDE surface. We used QGIS 3 software (http://www.qgis.org) to perform these analyses.

### Meteorological Factors, Mosquito Abundance and Infection Rate

We retrieved daily maximum temperatures and precipitations data from the ECA&D dataset (19) and extracted the data for every surveyed trap site in 2018. We aimed to estimate the association of meteorological parameters with the number of sampled *Culex* mosquitoes and with the infection rate (namely the proportion of WNV-positive mosquitoes). Since the effect of meteorological parameters on the outcomes of interest might be delayed (lag-effect), we focused on the meteorological parameters recorded up to 4 weeks (lag 1–4) before the night of sampling. Starting from the day before the sampling, we computed the weekly average of maximum temperatures and the weekly number of wet days (number of days in a week with precipitation > 0.5 mm).

Analyses were performed in the framework of the generalized linear mixed models (GLMMs) (20). Specifically, we applied a GLMM including a site-specific and week-specific random effect to account for site and temporal heterogeneity. The abundance of mosquitoes sampled per trap and night was analyzed by applying a linear mixed model with a normal distribution and identity link function. We used the natural logarithm of the count of sampled mosquitoes as the dependent variable and the meteorological parameters recorded up to 4 weeks before the sampling as predictors (lag 1–4). The per trap and night infection rate was analyzed by applying a generalized linear mixed model with Poisson distribution and log link function. We used the estimate of infected mosquitoes as the dependent variable, the number of sampled mosquitoes as offset and the meteorological parameters recorded up to 4 weeks before the sampling as predictors (lag 1–4). The association between temperatures and outcomes was assessed by estimating the coefficient for a unit increase (1°C) in the weekly average of maximum temperatures. The association between precipitations and outcomes was assessed by estimating the coefficient for a unit increase (1 day) in the weekly number of wet days. Analyses were performed with R software (*lme4* package).

## Results

### Mosquito Results

385,293 mosquitoes belonging to 13 species were sampled between June 4 and October 25, (Table 2). Mosquitoes of the *Culex* genus were grouped into 2,337 pools, of which 232 tested positive for lineage 2 of the WNV. The vast majority of tested pools and all pools which tested positive were composed of *Culex (Cx.) pipiens* (Table 3). The 134 seasonal traps collected 269,952 *Cx. pipiens* specimens; of these, 266,854 were tested in 2,254 pools, and 226 were WNV-positive. From among the 134 seasonal traps, 85 collected at least one WNV-positive pool (Figure 1). A total of 1,101 mosquitoes were sampled in the 9 extra traps activated in cities (Bologna, Modena, and Ferrara) in August. All mosquitoes from these samples (823 *Cx. pipiens*, 171 *Aedes (Ae.) albopictus*, and 107 *Ae. caspius*) were tested, and 6 out of the 14 *Cx. pipiens* pools resulted positive. No positive pools were detected in the other extra traps only activated for part of the season, mainly in northern Lombardy. In order to evaluate the possible involvement of *Ae. caspius* in virus circulation, we also tested 160 selected pools of this species (for a total of 8,211 specimens) collected between July 17 and August 14. All gave negative results.

**Table 2:** Mosquitoes sampled during the 2018 surveillance season.

**Species**	**Emilia-Romagna (%)**	**Lombardy (%)**	**Extra traps**	**Total (%)**
*Ae. albopictus*	4557 (1.6)	1743 (1.7)	665 (19.3)	6965 (1.8)
*Ae. berlandi*	1 (<0.5)			1 (<0.5)
*Ae. caspius*	43526 (15.7)	45299 (43.4)	118 (3.4)	88943 (23.1)
*Ae. cinereus*	3 (<0.5)			3 (<0.5)
*Ae. geniculatus*	15 (<0.5)			15 (<0.5)
*Ae. vexans*	7634 (2.8)	606 (0.6)	412 (11.9)	8652 (2.2)
*An. maculipennis s.l*.	753 (<0.5)	7007 (6.7)	1 (<0.5)	7761 (2.0)
*An. plumbeus*	10 (<0.5)	4 (<0.5)		14 (<0.5)
*Cq. richiardii*	658 (0.2)			658 (0.2)
*Cs. annulate*	3 (<0.5)	32 (<0.5)		35 (<0.5)
*Cs. longiareolata*	1 (<0.5)			1 (<0.5)
*Cx. modestus*	35 (<0.5)			35 (<0.5)
*Cx. pipiens*	220255 (79.4)	49697 (47.6)	2258 (65.4)	272210 (70.7)
Total	277451	104338	3454	385293

**Table 3:** Tested and WNV-positive mosquito pools for the 2018 surveillance season.

		**E-R**			**Lom**			**Extra-traps**			**Total**	
**Species**	**N**	**Pools**	**pos**	**N**	**pools**	**pos**	**N**	**pools**	**pos**	**N**	**pools**	**pos**
*Ae. albopictus*				16	2		177	11		193	13	
*Ae. caspius*	8211	160		5	1		107	5		8323	166	
*Cx. modestus*	35	6								35	6	
*Cx. pipiens*	220255	1554	194	46599	700	32	2258	77	6	269112	2331	232
Total	228501	1720	194	46620	703	32	2542	93	6	277663	2516	232

The average of collected mosquitoes per sample at provincial level showed the highest values in the western part of Emilia-Romagna, in Piacenza, Reggio Emilia, and Parma provinces (Table 4). A significant difference in the number of collected mosquitoes was recorded between Lombardy and Emilia-Romagna traps (considering only seasonal traps: μ_E−R =_ 258, CI 233-283, μ_LOM =_ 135, CI 110-159, *t* = 5.9016, *p* = 4.6e-9).

**Table 4:** Details of entomological surveillance at provincial level during the 2018 surveillance season.

**Region**	**Prov**.	**First WNV/+ pool**	**Last WNV/+ pool**	**Days**	Average sampled^*^	**CI**	**Total sampled**	**Est. positive mosq**.	**WNND cases**
Emilia-Romagna	BO	19/6	7/9	80	227	180-273	30603	49	41
	FC	5/7	2/8	28	103	38-168	2780	2	2
	FE	19/6	6/9	79	248	201-295	61730	89	14
	MO	3/7	14/8	42	243	182-303	25864	39	23
	PC	10/7	2/10	84	380	292-468	37619	8	2
	PR	17/7	28/8	42	303	202-404	24257	14	1
	RA	24/7	21/8	28	124	88-160	6574	5	13
	RE	15/6	24/8	70	334	241-428	30067	25	5
	RN	31/7	31/7		88	22-154	1584	1	0
Lombardy	BS	26/7	23/8	28	185	115-255	13811	3	1
	LO	2/8	2/8	0	152	70-234	4103	1	1
	MI	7/8	6/9	30	62	38-85	2895	2	6
	MN	17/7	20/8	34	164	98-230	14047	14	4
	PV	30/7	20/8	21	175	120-230	11919	17	1
	BG				40	16-63	1472	0	0
	CO				−		20	0	1
	CR				92	38-147	2444	0	2
	LC				57	12-102	341	0	0
	MB				6	2-10	48	0	0
	VA				−		32	0	0

* *In traps which work throughout the season*.

### Characterization of Virus Circulation

**Figure 2:**
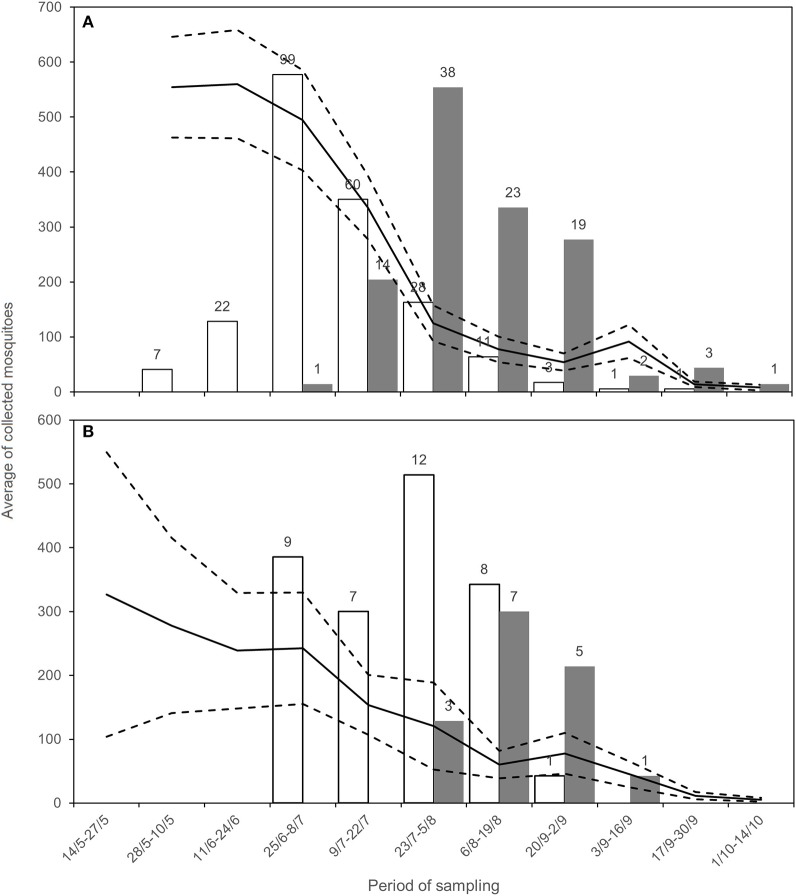
Average of sampled *Cx. pipiens* mosquitoes (black line) with CI (dashed lines), positive estimated mosquitoes (white, number above the bar) and number of WNND cases (gray, number above the bar) for Emilia-Romagna **(A)** and Lombardy **(B)**, during the surveillance.

In 2018, WNV circulation was first detected on June 15 in Emilia-Romagna and on July 17 in Lombardy. We collected the last positive pools on September 6 and October 2 in Lombardy and Emilia-Romagna, respectively (Table 4). The epidemic curve of WNND human cases was postponed with respect to the detection of positive mosquitoes (Figure 2).

**Figure 3:**
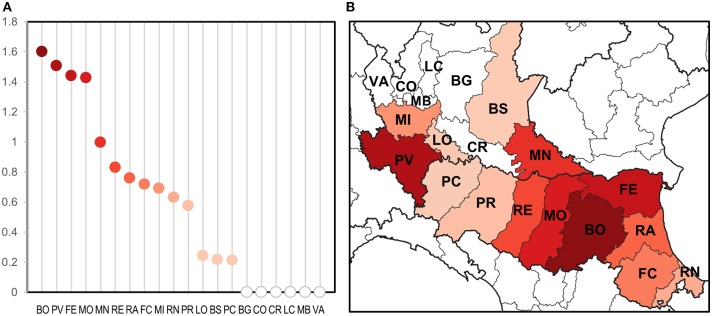
Evaluation of WNV circulation intensity according to number of estimated positive mosquitoes on 1,000 sampled mosquitoes at provincial level **(A)** and the same on a map **(B)**.

Only taking seasonal trap data into account, we evaluated the intensity of circulation at the provincial level considering the number of estimated WNV positive mosquitoes on total mosquitoes tested (Figure 3). This highlighted groups of provinces with a different circulation intensity: (i) a group of provinces with a more intense circulation (including central Emilia-Romagna and Pavia); (ii) a group of provinces with an intermediate circulation intensity (in the central and eastern surveillance zones); (iii) a group with a low circulation intensity (Lodi, Brescia, and Piacenza); and (iv) a group in which the virus was undetected (northern Lombardy). Provinces with a more intense circulation often recorded an early and more prolonged virus circulation (Table 4), and a major number of WNND cases.

**Figure 4:**
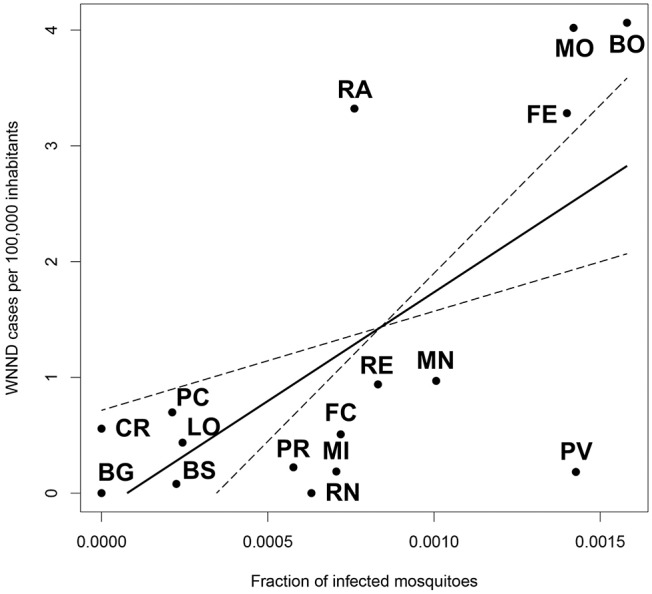
Linear model showing the relationship between the fraction of infected mosquitoes at provincial level, and the incidence of WNND cases in the same province.

We found the existence of a significant relationship between the rate of infected mosquitoes collected in each province and the incidence of WNND cases in the same province (*p* = 0.0013). Specifically, we estimated that an increase of 1%0 in the rate of WNV positive pools leads to an additional 1.88 WNND cases per 100,000 inhabitants (Figure 4).

### Relationship Between the Number of Collected Mosquitoes and WNV Incidence

**Figure 5:**
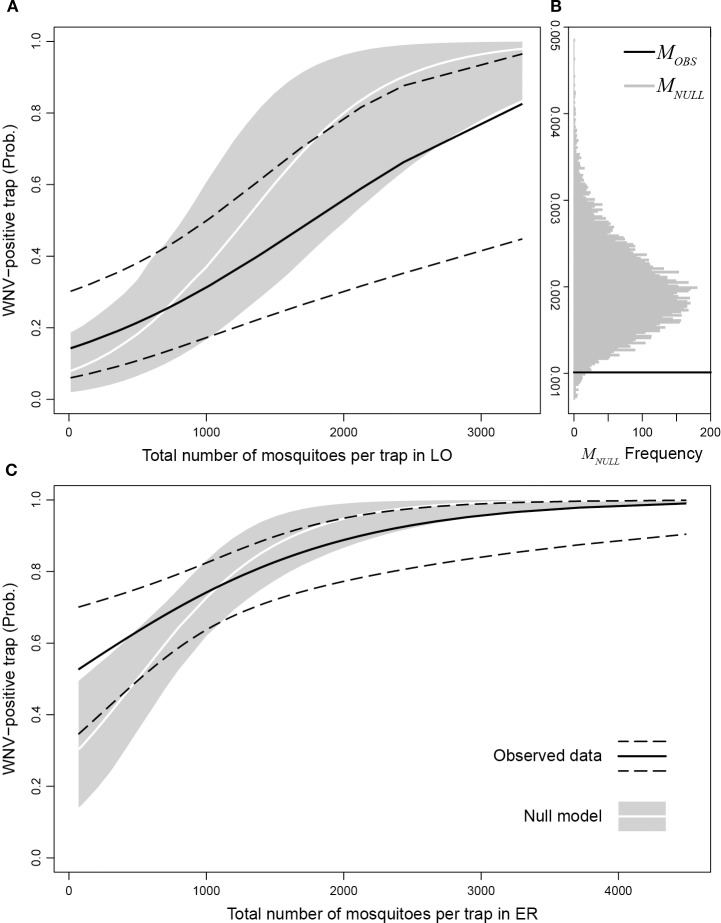
Probability of observing a WNV-positive trap as a function of the number of mosquitoes per trap in Lombardy **(A)** and Emilia-Romagna **(C)** estimated through observed data (black solid lines: best fit, dashed lines: 95% confidence interval) compared with the null model (white lines: median, gray areas: 95% interval). **(B)** distribution of the marginal increase in the probability to observe a WNV-positive trap due to an additionally caught mosquito obtained with the null model (*M*_*NULL*_, gray bars) compared to *M*_*OBS*_ (black line).

We found a significantly higher probability of finding WNV-positive traps in Emilia-Romagna with respect to Lombardy (*p*-value = 8.7e-5). We also estimated the marginal increase in the probability of observing a WNV-positive trap due to an additional mosquito caught as *M*_*OBS*_ = 0.00102 (*p*-value = 0.0021). Figure 5B shows the distribution of the marginal increase in the probability of observing a WNV-positive trap due to an additional mosquito caught obtained with the null model (*M*_*NULL*_, gray bars) compared to *M*_*OBS*_ (black line). We found that *M*_*OBS*_ is significantly lower than *M*_*NULL*_ (*p*-value = 0.0232), this means that real number of captured mosquitoes affects less than in the simulations the probability to detect WNV in a trap, suggesting that mosquito abundance in a given site provides a dilution effect on WNV circulation. Figure 5 displays the probabilities of observing a WNV-positive trap as a function of the number of mosquitoes per trap in Lombardy (panel A) and Emilia-Romagna (panel C), estimated through the observed data (black solid lines: best fit, dashed lines: 95% confidence interval) and the null model (white lines: median, gray areas: 95% interval). Panels A and C in Figure 5 show that the probability of finding WNV-positive traps is higher than expected when the abundance of caught mosquitoes is low (i.e., black lines are higher than the white lines), while the opposite is true when the abundance of caught mosquitoes is high (i.e., black lines are lower than the white lines).

Despite the probability of finding that WNV increases with the number of mosquitoes (i.e., *M*_*OBS*_ > 0), the probability of a single mosquito being infected decreases as the number of collected mosquitoes increases. Thus, the estimated probability of a single mosquito collected in one site being infected decreased with the increase in the number of collected mosquitoes in the same site.

**Figure 6:**
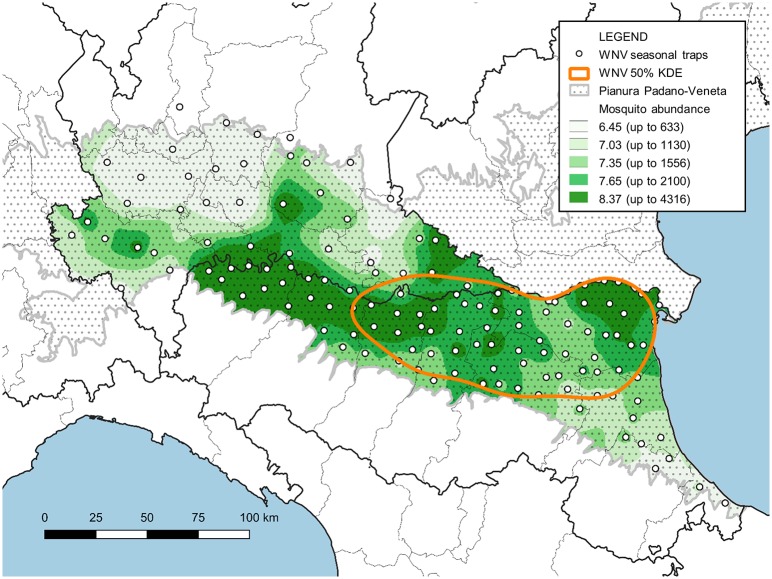
Map of the surveyed area with the IDW interpolation of *Cx. pipiens* females collected between June and August (expressed as natural logarithm) and the hot-spot of WNV circulation (represented by 50% of the KDE of sites with at least one positive pool).

Fifty percent of the positive traps' KDE revealed a single hot spot in Emilia-Romagna where WNV circulation is more intense (in orange in Figure 6). Overlaying this map onto the map of sampled mosquitoes, directly related to mosquito abundance, highlighted that areas where mosquitoes are most abundant are not necessarily included in this WNV hot spot (Figure 6).

### Association Between Weather Data and WNV Circulation

**Figure 7:**
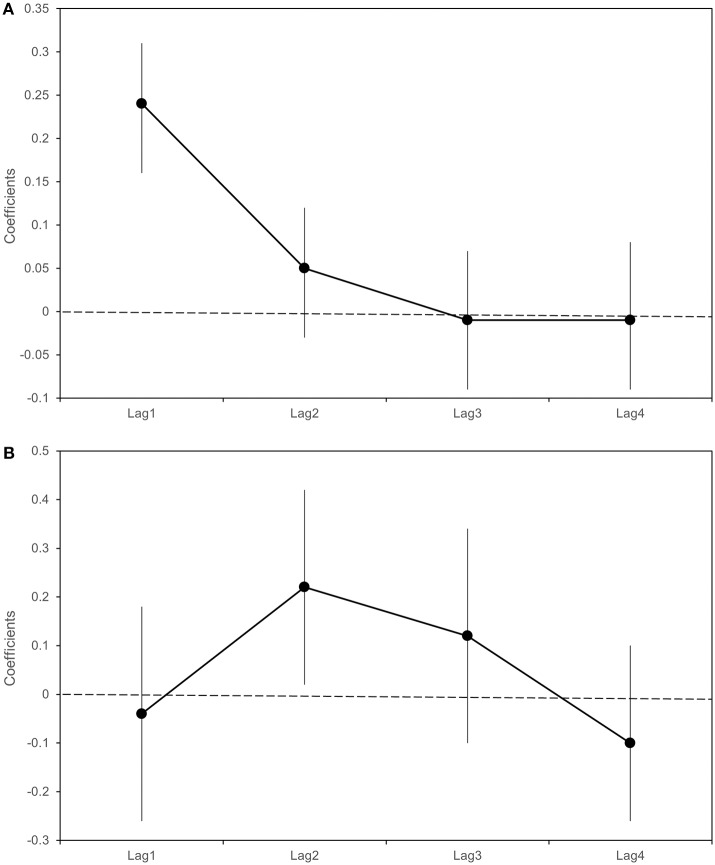
Beta coefficients (black dots) and 95% Confidence intervals (bars) with respect to a 1°C increase in weekly average maximum temperatures in the previous 4 weeks (lag 1–4). Linear regression estimates for the logarithm of the number of sampled *Culex pipiens* **(A)**; Poisson regression estimates for Infection Rate **(B)**.

Table 5 and Figure 7 report the β coefficients and 95% CI for the fixed effects of lagged meteorological parameters. Fixed effect coefficients are average estimates from among all sampled traps of the association between a unit increase in the meteorological parameters and the outcomes. β coefficients are estimated under the null hypothesis of absence of association (β = 0), thus a positive estimate of β coefficient suggests that an increase in the meteorological parameter is associated with an increase in the outcome under study (number of sampled mosquitoes or infection rate).

**Table 5:** Linear regression coefficient (β) and 95% Confidence Interval (CI) for the logarithm of the number of *Culex pipiens* Sampled and Lagged Meteorological Parameters.

**Parameter**	**β^1^**	**95% IC**
**1°C Increase in weekly average maximum temperatures**		
Lag1	**0.24**	**0.16 – 0.31**
Lag2	0.05	−0.03 – 0.12
Lag3	−0.01	−0.09 – 0.07
Lag4	−0.01	−0.09 – 0.08
**1 Day increase in the weekly number of wet days**		
Lag1	0	−0.10 – 0.10
Lag2	−0.06	−0.13 – 0.01
Lag3	−0.01	−0.08 – 0.06
Lag4	−0.04	−0.11 – 0.03

1 *Linear regression coefficient (β): it indicates the average change in the logarithm of Cx. pipiens sampled associated with a 1°C increase in weekly average maximum temperatures and 1 day increase in weekly number of wet days. p value < 0.05 in bold*.

As shown in Table 5 and Figure 7, we found an association between the logarithm of the number of sampled mosquitoes and the weekly average of maximum temperatures recorded the week before sampling (β at lag1 : 0.24, 95% CI 0.16–0.31). In contrast, we did not find evidence of association between the logarithm of the count of sampled mosquitoes and the number of wet days at any lag (Table 5).

When analyzing the proportion of WNV positive mosquitoes, we observed a positive association between the infection rate and the weekly average of maximum temperatures recorded two weeks before the sampling (β lag 2: 0.22; 95% CI: 0.02-0.42) (Table 5). No evidence of association was detected between the infection rate and the weekly number of wet days observed in the previous 4 weeks (Table 6).

**Table 6:** Poisson regression coefficient for Infection Rate and Lagged Meteorological Parameters.

**Parameter**	**β^1^**	**95% IC**
**1°C Increase in weekly average maximum temperatures**		
Lag1	−0.04	−0.26−0.18
Lag2	**0.22**	**0.02**—**0.42**
Lag3	0.12	−0.10−0.34
Lag4	−0.1	−0.26−0.10
**1 Day increase in number of wet days**		
Lag1	0.07	−0.17−0.32
Lag2	0.13	−0.06−0.32
Lag3	−0.18	−0.37−0.02
Lag4	0	−0.16−0.16

1 *Poisson regression coefficient (β): the exponential of β indicates the rate ratio, namely the change in the infection rate (multiplicative term) associated with a 1°C increase in weekly average maximum temperatures and 1 day increase in weekly number of wet days. p value < 0.05 in bold*.

## Discussion

In 2018, in Italy as in other European countries (4), the WNV transmission season began earlier than in previous years. In fact, in Emilia-Romagna, the season started earlier by about one month with respect to previous transmission seasons and the Lombardy region. Circulation intensity and the number of human cases were also the highest observed since the first appearance of WNV in Italy. Starting from the central Provinces of Emilia-Romagna (Modena, Bologna, and Ferrara), WNV circulation progressively extended to both the western and eastern part of the surveyed area covering all the Emilia-Romagna region right up to Lombardy (Pavia, Brescia, Milano, Lodi, and Mantua). However, the virus was undetected in mosquitoes collected in Cremona, Bergamo, Monza Brianza, Varese, and Lecco. The early detection of the virus circulation at provincial level makes it possible to start test blood bags when an area is affected, avoiding infected transfusions.

Reported data confirmed the *Cx. pipiens* mosquito as the main vector of WNV in the surveyed area. To evaluate the involvement of other species, pools of *Ae. caspius* and *Ae. albopictus* were also tested during this survey, without any positive results. The possible involvement of *Ae. albopictus* as a bridge vector in the WNV cycle was deduced due to WNV positive pools collected in the field in the U.S. (21) and Turkey (22), and the vectorial competence demonstrated in experimental studies (23, 24). The possible vector role of *Ae. caspius*, since WNV-positive pools of this mosquito were collected in the field in the surveyed area (25), still remains to be demonstrated as we have no clues as to these two mosquitoes' involvement in the WNV cycle in our epidemiological scenario. Independently of intrinsic competence, this was likely due to the host preference of these mosquitoes which, contrary to *Cx. pipiens* (strictly ornithophilic), are mammophilic feeders. Also, *Cx. modestus*, a species more competent for WNV than *Cx. pipiens* in a laboratory study (26), had a marginal role in the monitored area's WNV cycle. This is due to the scarcity of this species, which is strictly linked to the natural environment (13). Results of extra-plan samples in some cities highlighted the intensive viral circulation in 2018, also involving urban areas which are usually less suitable than rural areas for WNV circulation. It is likely that virus circulation in urban areas, also sustained by *Cx. pipiens*, had an important role in causing the large amount of human cases recorded in 2018.

The surveillance system showed a relevant difference in WNV circulation intensity between the two regions, which was also confirmed by the difference in the number of human cases. Nevertheless, different mosquito sampling efforts between the two regions, and the difference recorded in the GLM, was largely ascribable to the different circulation extent between the two areas. The applied models demonstrate the final number of infected mosquitoes increases when the number of mosquitoes also increases. However, the probability of a single mosquito being infected decreased with mosquito abundance, revealing a non-linear relationship between mosquito abundance and virus circulation. This result agreed with the absence of a direct correlation between mosquito abundance and virus circulation in years with a lower WNV circulation (7). The geographic interpolation of the data utilized to characterize this relationship demonstrated that the rate of infected mosquitoes was not higher where mosquito density was highest. This confirms a less than linear correlation between virus presence and mosquito abundance, temporally and spatially.

The data obtained demonstrated the obvious importance of mosquito abundance but highlighted that this is not the limiting factor for virus circulation in the considered area, which we identified as likely in the number of infected hosts. If we postulate the finite number of infected hosts as a limiting factor, and then the finite capacity to attract mosquitoes, we can hypothesize a dilution effect exerted by the mosquitoes not attracted to them, above a certain number of mosquitoes. In this theoretical scenario, the synchronization of mosquito population peaks and WNV-susceptible birds is fundamental in determining WNV dynamics and the environmental viral load for the season. The observed pattern may also support the hypothesis that increased mosquito density leads to increased avian defensive behaviors, leading to a shift in mosquito feedings toward less defensive hosts, such as mammals which are not WNV-susceptible (27, 28).

A complex interaction between mosquito abundance and susceptible hosts determines the level of virus circulation, which differed in direct relation (namely more mosquitoes, more virus) and is usually retained in vector-borne diseases. We recommend that this result is considered in the construction of epidemiological models and in the cost-efficacy analysis of vector control methods.

In this study, temperature was confirmed as one of the most important factors in determining the virus circulation. We detected an association between temperatures recorded one week and two weeks before mosquito sampling and the mosquito infection rate. Temperature influenced the bionomics of mosquitoes in several ways, for example, by increasing vectorial capacity or shortening the extrinsic incubation period and development times (29). These results are consistent with previous studies that observed a positive association between temperatures and WNV-positive mosquitoes and human cases observed in the following weeks (30–32). This association, already confirmed by several research studies in different ecological settings, is alarming in a global warming scenario, since it implies that increasing temperatures will increase the risk of WNV circulation. The same association can partially explain the recent emergence of the virus, which could be linked to the increase in temperatures recorded in recent years in the surveyed area (33). Interestingly, we observed the difference of one or two weeks in the temperature's ability to influence the number of mosquitoes and the virus infection rate. This observed delay could be explained with the virus' extrinsic incubation period, which consistently lasts about one week at temperatures recorded in the surveyed area (34, 35).

As recorded in another study (31), we were unable to find a consistent link between rainfall (expressed as wet days), mosquito abundance and the mosquito infection rate. An issue related to this process, which can hide the effects of rain, could be the background noise linked to the fall in temperature caused by rainfall. However, water availability under ordinary conditions was not a limiting factor in the study area (due to the widespread presence of irrigating networks, rivers, and water basins).

A human case of WNND is of striking relevance, due to the severity of the symptoms associated with the infection. However, occurrence is rare and not relevant for the environmental persistence of the virus. Despite this, occurrence of WNND was the best available indicator of circulation intensity in people, since there was no organic and standardized system for WNV fever diagnosis. The timing and location of WNND cases are random due to the low rate of WNND cases in infected persons (<1%), the complexity in defining a certain site of patient infection and has human density as one of the main determinants (36), in a particular area. Despite this, we were able to find a clear relationship between the rate of positive mosquitoes and human cases, highlighting the surveillance ability to assess the intensity of virus circulation in a given area at an early stage.

The entomological monitoring described in this work is part of a multidisciplinary surveillance integrating other monitoring systems (wild bird testing and syndromic surveillance of horses). Entomological surveillance often provided the first sign of WNV circulation and, due to the sampling program's standardized regularity, allowed for a fine characterization of the period, area and intensity of circulation. This provides essential data for modulating actions to limit the health risks associated with the circulation of WNV.

## Data Availability Statement

The raw data supporting the conclusions of this article will be made available by the authors, without undue reservation, to any qualified researcher.

## Author Contributions

MCa, PA, RC, DC, MPC, MCh, GG, MT, RB, and DL conceived and designed the surveillance system. MCa, SC, and TT conducted the sampling and mosquito identification. PB and DT performed the molecular analysis. MCa, GM, LB, and AA processed the data and performed statistical analyses. MCa wrote the manuscript. All authors reviewed and approved the final manuscript.

## Conflict of Interest

The authors declare that the research was conducted in the absence of any commercial or financial relationships that could be construed as a potential conflict of interest.
